# The Geometry of Noise in Color and Spectral Image Sensors

**DOI:** 10.3390/s20164487

**Published:** 2020-08-11

**Authors:** Axel Clouet, Jérôme Vaillant, David Alleysson

**Affiliations:** 1 CEA, Univ. Grenoble Alpes, LETI 38054 Grenoble CEDEX 9, France; jerome.vaillant@cea.fr;; 2 Laboratoire de Psychologie et NeuroCognition, CNRS UMR 5105 Grenoble, France; david.alleysson@univ-grenoble-alpes.fr

**Keywords:** image, sensor, color, multispectral, noise, geometry

## Abstract

Digital images are always affected by noise and the reduction of its impact is an active field of research. Noise due to random photon fall onto the sensor is unavoidable but could be amplified by the camera image processing such as in the color correction step. Color correction is expressed as the combination of a spectral estimation and a computation of color coordinates in a display color space. Then we use geometry to depict raw, spectral and color signals and noise. Geometry is calibrated on the physics of image acquisition and spectral characteristics of the sensor to study the impact of the sensor space metric on noise amplification. Since spectral channels are non-orthogonal, we introduce the contravariant signal to noise ratio for noise evaluation at spectral reconstruction level. Having definitions of signal to noise ratio for each steps of spectral or color reconstruction, we compare performances of different types of sensors (RGB, RGBW, RGBWir, CMY, RYB, RGBC).

## 1. Introduction

Images acquired by the sensor in digital cameras need to be processed for being displayed on an output screen. But because of the random absorption of photons, image capture is always subject to noise. Noise can be amplified by the processing operations, the amplification at the output image depends both on physical properties of the sensor (spectral sensitivities, dynamic range, etc.) and the kind of processing applied to the raw image [1,2,3,4,5].

In the literature, one common problem is to find the best hardware/software configuration to minimize the amount of noise of the output image [6]. The question of sensor optimization is very complex, because it is a spatio-spectral inverse problem [7]. The problem involves the choice of spectral sensitivities associated to color channels and their distribution on the color filter array (CFA), for optimizing both color rendering and spatial quality.

**Figure 1:**
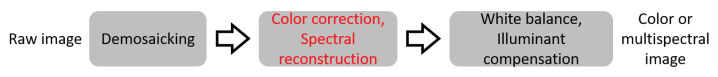
Common linear processing chain in an imaging system.

Here, we assume the whole processing chain is linear. Under this linearity hypothesis, the overall processing can be split into several linear steps, shown in Figure 1. All these linear steps can be optimized independently to minimize noise based on least square error. Mean square error optimization was shown to work well for the demosaicking step [7,8], which is not considered in this study. In this paper, we focus on the problem of the choice of spectral sensitivities and its impacts on noise amplification in the output image.

Usual denoising block is voluntarily discarded from this processing chain because our aim is to test noise amplification based on several sensor designs. White balance is not considered either because its noise amplification could be controlled. The only processing step under study in this paper is color correction. Therefore, we consider that each pixel in the raw image contains a vector of raw values (triplet in case of RGB sensor). Color correction is a pixel-wise transformation that converts raw data into displayable RGB triplets. It is actually composed of two operators, a spectral reconstruction operator [9] followed by an operator that projects spectral data onto the display space [10,11].

We investigate noise amplification through color correction by using a geometrical framework to represent signals and noise at the spectral and color reconstruction steps. Algebra and geometry for sensor acquisition has already been stated [10,12]. The representation we use here considers inter-channel spectral correlations because sensor basis are always non-orthogonal. Consequently, we introduce contravariant signal to noise ratio to take in consideration this sensor’s metric. This one is then appropriate to determine noise amplification at spectral reconstruction step.

## 2. Example of Noise Amplification through Color Correction

**Figure 2:**
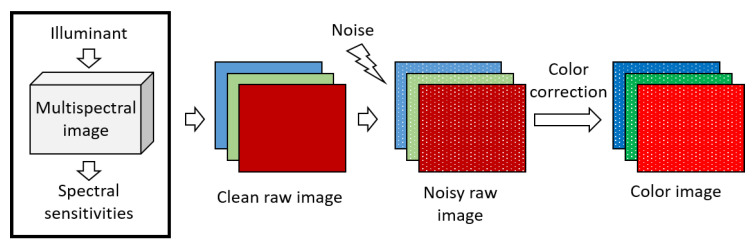
Simulation framework to display raw noisy and corrected images.

To illustrate the problem of noise amplification we start implementing some simulations based on the high resolution multispectral images from the ReDFISh dataset [13]. The simulation framework is given in Figure 2.

It uses reflectance data ($\rho_{p,q}\left(\lambda \right)$, p,q the pixel location) from multispectral images to compute raw frames independently for each channel. These frames are then stacked such that each pixel of the obtained image contains the information of all channels without performing any demosaicking.

Simulations are performed using Python. They start by choosing an illuminant whose spectral distribution is denoted $I_{1lux}\left(\lambda \right)$ and hypothetic spectral sensitivities of a sensor denoted $Q_{i}\left(\lambda \right)$. If the sensor is RGB, i={r,g,b}. Raw signals are then computed [14,15] by applying this equation on each pixel for each channel:

(1) $$\begin{array}{cc} \langle S_{p,q,i}\rangle & =K.\int_{-\infty }^{\infty }I_{1lux}\left(\lambda \right).\rho_{p,q}\left(\lambda \right).Q_{i}\left(\lambda \right).d\lambda \\ K & =\frac{CVF.N_{lux}.t_{i}.a_{pix}^{2}}{4f_{\#}^{2}} \end{array}$$

*K* is an exposure factor depending on several parameters such as pixel size ($a_{pix}$ in m), the aperture of the objective lens ($f_{\#}$) the integration time ($t_{i}$ in s), an illumination level ($N_{lux}$ in lux) and finally the CVF, a conversion factor between electron values and digital units (bits) [14]. 〈〉 is an average operator used to denote noise free signals. After stacking the raw planes, we get a noise free raw image.

Then photon shot noise and readout noise are added to the raw image using random functions from Numpy library of Python. Photon shot noise has a Poisson distribution and its variance $\sigma_{ph}^{2}$ is equal to the mean signal value. Readout noise has a Gaussian distribution, its standard deviation $\sigma_{rn}$ is often given in sensor datasheets as an electron quantity. Noisy image is obtained by:

(2) $$S_{p,q,i}=\langle S_{p,q,i}\rangle +\sigma_{ph}\left(\langle S_{p,q,i}\rangle \right)+\sigma_{rn}$$

Photon Shot noise is pixel dependent, this dependency is denoted with parenthesis brackets. In these simulations, data are clipped between 0 et 1 and computed in double type so signal quantization is not considered. Other sources of noise are also neglected in this paper.

**Figure 3:**
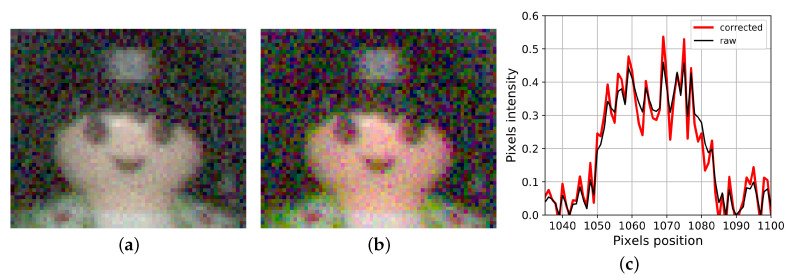
(**a**) Noisy raw image, (**b**) Image after color correction. (**c**) Intensity profile of the green channel at the horizontal median.

As an example of noise amplification we consider spectral sensitivities of a classical RGB sensor under constant photon flux illuminant and experiment parameters of Table 1. Then, we compute a noisy raw image and apply a color correction matrix (CCM) to display Figure 3.

A challenge for many authors is to find a way to reduce this amplification. This can be done by optimizing spectral sensitivities of the sensor [16,17,18] or by changing the way CCM is computed (adding regularization, tuning the CCM coefficients...) [9,19]. Complementary to these studies, the goal of our paper is mainly to describe the algebraic mechanisms that lead to this noise amplification.

## 3. Geometrical Representation of Sensors Signal and Noise

This section aims to display a geometry of signals and noise propagation from the noisy raw acquisition through spectral reconstruction and color correction. Cohen provided an interpretation of spectral and color acquisition by considering the metric of the sensor space (the angle between each axis represents the correlations between them) [10]. In his formalism, spectra are represented as vectors in the sensor space. Moreover, some authors represented noise in color spaces like CIELAB or CIE xyY [2,20]. The analysis of a noisy image is, here, represented as a cloud of points or a geometrical uncertainty domain around the signal mean value. We join these two approaches to represent noise as an uncertainty domain in Cohen’s formalism which considers the metric of the sensor. For visualization purposes and to illustrate the tendencies of noise domain evolution across processes, we set noise with a uniform statistical distribution.

Spectral sensitivities are continuous physical functions of wavelength which can be considered as vectors in a Hilbert space [21]. Inside that vector space, signals acquired from input light have a geometrical representation. For illustration we consider a sensor with two channels (red and blue). This two-dimensional representation can be extended to a *p*-dimensional vector space without loss of generality. So the model addresses multicolor (even multispectral) sensors having three color channels and more.

The considered Hilbert space owns a scalar product denoted 〈.|.〉 such that for two vectors $L_{1}$ and $L_{2}$:

(3) $$\langle L_{1}|L_{2}\rangle =\int_{-\infty }^{\infty }L_{1}\left(\lambda \right).L_{2}\left(\lambda \right).d\lambda$$

The . operator is the point-wise product between the two curves. The definition of this scalar product allows to represent the spectral sensitivities of the sensor, denoted $Q_{r}$ and $Q_{b}$ for our example. Their $L_{2}$ norms are derived from a square root of the scalar product of each considered vector with itself:

(4) $$\begin{array}{cc} {∥Q_{i}∥}_{2} & =\sqrt{\int_{-\infty }^{\infty }Q_{i}\left(\lambda \right).Q_{i}\left(\lambda \right).d\lambda }=\sqrt{\langle Q_{i}|Q_{i}\rangle }\\ i & =\{r,b\} \end{array}$$

The angle between the two sensitivity vectors [10] is computed as the scalar product divided by the product of norms.

(5) $$\cos\left(\theta_{rb}\right)=\frac{\langle Q_{r}|Q_{b}\rangle }{{∥Q_{r}∥}_{2}.{∥Q_{b}∥}_{2}}$$

**Figure 4:**
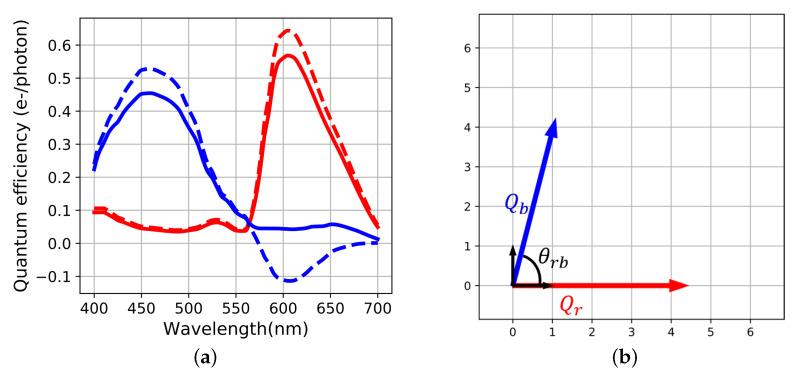
(**a**) Spectral sensitivities (continuous curves) and orthogonalized curves (dash curves, scaled by a factor five for visualization) (**b**) Corresponding geometrical representation. $\theta_{rb}=75^{\circ }$, the black arrows are the non-scaled vectors of the orthogonal basis.

The span of spectral sensitivity functions generates a vector subspace inside the Hilbert space of light spectra [10]. In practical case, these sensitivities are usually non-orthogonal because they have correlations between each other. To accurately display the sensor basis, we derived an orthogonal basis associated to the sensor spectral sensitivities using a Gramm–Schmidt process (Figure 4). Spectral sensitivities are linked to the orthogonal basis by a linear transform that could be considered for building the metric of the sensor space.

Considering a radiance vector *L* as the input light of the sensor, equations giving noise free signals (see Equation (1)) can be rewritten using a scalar product in Hilbert space of lights:

(6) $$\begin{array}{cc} S_{r} & =K\int_{-\infty }^{\infty }L\left(\lambda \right).Q_{r}\left(\lambda \right).d\lambda =K\langle L|Q_{r}\rangle \\ S_{b} & =K\int_{-\infty }^{\infty }L\left(\lambda \right).Q_{b}\left(\lambda \right).d\lambda =K\langle L|Q_{b}\rangle \\ L(\lambda ) & =I_{1lux}\left(\lambda \right).\rho \left(\lambda \right) \end{array}$$

To reduce the amount of notation, noise free signals are denoted without 〈〉 average operator.

If spectral sensitivities are sampled in a *n* number of wavelengths, these equations can be approximated in a vector way implying a matrix product [10,11,12]. This is a rectangle approximation of the integrals of Equation (6) given by:

(7) $$\left(\begin{array}{c} S_{r}\\ S_{b} \end{array}\right)\approx \underset{\tilde{K}}{\underbrace{K.\Delta \lambda }}\underset{F^{T}}{\underbrace{{\left(\begin{array}{cc} Q_{r}\left(\lambda_{1}\right) & Q_{b}\left(\lambda_{1}\right)\\ \vdots & \vdots \\ Q_{r}\left(\lambda_{n}\right) & Q_{b}\left(\lambda_{n}\right) \end{array}\right)}^{T}}}\left(\begin{array}{c} L(\lambda_{1})\\ \vdots \\ L(\lambda_{n}) \end{array}\right)=\tilde{K}.F^{T}.L$$

To represent raw data in the sensor’s space, we use the mathematical link between scalar product and orthogonal projection [22]. Each orthogonal projection of *L* on $Q_{i}$ (i={r,b}) can be expressed as a vector denoted $L_{Q_{i}}$ collinear to $Q_{i}$. The $L_{2}$ norm of this vector is denoted ${\tilde{S}}_{i}$. This norm can be derived from the scalar product between *L* and $Q_{i}$ which can be rewritten using the raw signals $S_{i}$:

(8) $$\begin{array}{cc} L_{Q_{i}} & =\frac{\langle L|Q_{i}\rangle }{{∥Q_{i}∥}_{2}^{2}}.Q_{i}=\frac{1}{\tilde{K}}.\frac{S_{i}}{{∥Q_{i}∥}_{2}^{2}}.Q_{i}\\ {\tilde{S}}_{i} & =\frac{1}{\tilde{K}}.\frac{S_{i}}{{∥Q_{i}∥}_{2}}={∥L_{Q_{i}}∥}_{2}\\ i & =\{r,b\} \end{array}$$

${\tilde{S}}_{i}$ may be misplaced in an uncertainty domain because of raw data noise. The noise level which affects ${\tilde{S}}_{i}$, denoted ${\tilde{\sigma }}_{i}$, is computed using the same transform applied on $\sigma_{i}$:

(9) $${\tilde{\sigma }}_{i}=\frac{1}{\tilde{K}}.\frac{\sigma_{i}}{{∥Q_{i}∥}_{2}}$$

**Figure 5:**
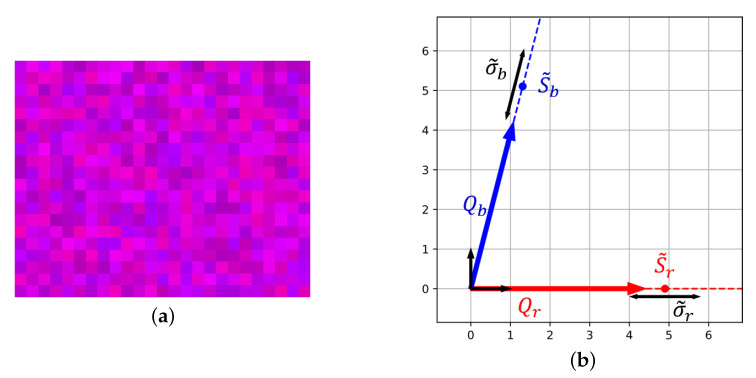
(**a**) Visual results of raw acquisition with superposed red and blue channel, (**b**) locations of the raw points and raw noise segments in the sensor space ${\tilde{S}}_{i}$ and ${\tilde{\sigma }}_{i}$.

For illustration, a simulation of raw acquisition over 500 pixels is computed in Figure 5. The chosen input radiance *L* is constant photon flux along wavelength and *K* is set such that acquisitions are quiet noisy (K=1.385 S.I units). The levels of raw noises $\sigma_{r}$ and $\sigma_{b}$ are computed as in Equation (2), but for visualization purposes, their statistical distribution are set as uniform.

Raw signals are now processed to perform spectral reconstructions from sparse raw values. In the following, we give a geometrical representation of signal and noise for three different linear methods of spectral reconstructions. In a first method, called intrinsic reconstruction, the reconstruction operator is calculated from the sensor sensitivities only. In a second method a database of spectra is used as a training set to build the reconstructing operator with a linear regression. In the third method we use Tikhonov regularization. Finally, we show that these operators can be considered as part of the color correction matrix whose geometrical interpretation is also depicted.

All along this section, one may keep in mind that any point of the sensor plane (see Figure 4b) is a linear combination of $Q_{r}$ and $Q_{b}$. Thus, each point is associated to a continuous function of wavelength similarly to $Q_{r}\left(\lambda \right)$ and $Q_{b}\left(\lambda \right)$.

### 3.1. Intrinsic Spectral Reconstruction (No Training Set)

Color and snapshot multispectral sensors classically contain few spectral channels, the dimension of acquired spectra is restricted to few numbers (*p* channels) compared to the number of variables (*n* wavelength samples) in the spectral domain. To retrieve spectra from acquired data we compute a reconstruction matrix called *R*. The most straightforward approach consists in performing a least square minimization between the n×n identity operator $I_{n}$ and $RF^{T}$. We get the reconstructing operator *R* given by:

(10) $$\begin{array}{cc} R & =argmin_{\hat{R}}\left({∥I_{n}-\hat{R}F^{T}∥}_{2}^{2}\right)\\ \Leftrightarrow R & =F{\left(F^{T}F\right)}^{-1} \end{array}$$

*R* is the pseudo-inverse matrix of *F* and reciprocally, the pseudo-inverse matrix of *R* is *F*. For our red and blue sensor, the spectral reconstruction of *L*, denoted $\hat{L}$, using this approach can be written as in [10,11]:

(11) $$\begin{array}{cc} \hat{L} & =\frac{1}{\tilde{K}}R\left(\begin{array}{c} S_{r}\\ S_{b} \end{array}\right)=F{\left(F^{T}F\right)}^{-1}F^{T}L \end{array}$$

**Figure 6:**
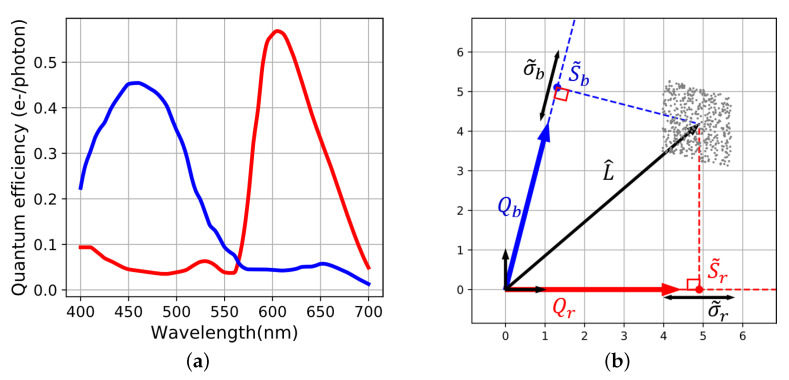
(**a**) Spectral sensitivities of the sensor, (**b**) Noisy intrinsic spectral reconstruction (without training set), the positions of reconstructed spectra from pixels of Figure 5 constitute the cloud of points. $\hat{L}$ is the position of the noise free spectral reconstruction. Its orthogonal projections on $Q_{r}$ and $Q_{b}$ is ${\tilde{S}}_{r}$ and ${\tilde{S}}_{b}$. The size of the noise parallelogram is given by ${\tilde{\sigma }}_{r}$ and ${\tilde{\sigma }}_{b}$.

The operator $F{\left(F^{T}F\right)}^{-1}F^{T}$ is an orthogonal projector [10]. So $\hat{L}$ is placed in the sensor’s plane such that its orthogonal projection on $Q_{r}$ and $Q_{b}$ are equal to ${\tilde{S}}_{r}$ and ${\tilde{S}}_{b}$ (Figure 6). Because of noise, the location of $\hat{L}$ is inside an uncertainty area whose extension is driven by ${\tilde{\sigma }}_{r}$ and ${\tilde{\sigma }}_{b}$. Figure 6b shows the location of the noise free $\hat{L}$ surrounded by the points computed from the noisy simulation of Figure 5. They are distributed in a parallelogram shape because of the choice of the uniform distribution of raw noise.

The area A of the parallelogram represents the level of noise of the spectral reconstruction. It depends not only on the raw noise levels, but also on the correlation between the channels through $\theta_{rb}$:

(12) $$A=\frac{\sigma_{R}.\sigma_{B}}{\sin(\theta_{rb})}$$

This angle decreases when correlation are higher making A wider. So for identical raw noise levels, the more correlated the channels are, the noisier the spectral reconstruction is.

### 3.2. Linear Regression over a Spectral Training Set

To increase estimation accuracy, it is usual to perform a linear regression over a training set of radiance spectra [18]. This training set can be written as a n×m matrix $T_{set}$ where *n* is the number of wavelengths and *m* the number of radiance spectra. Reconstructing operator based on training set is denoted $R_{t}$. It is computed performing a least square minimization between $T_{set}$ and its reconstruction from raw values:

(13) $$\begin{array}{cc} R_{t} & =argmin_{\hat{R_{t}}}\left({∥T_{set}-\hat{R_{t}}F^{T}T_{set}∥}_{2}^{2}\right)\\ \Leftrightarrow R_{t} & =T_{set}T_{set}^{T}F{\left(F^{T}T_{set}T_{set}^{T}F\right)}^{-1} \end{array}$$

**Figure 7:**
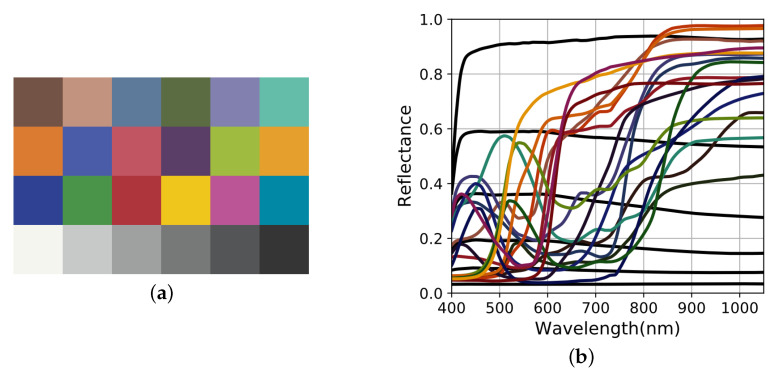
(**a**) Color rendering of X-Rite ColorChecker Classic. (**b**) Corresponding reflectance spectra.

For color applications, a well-known spectral dataset is the X-Rite ColorChecker Classic (Figure 7). Radiance of $T_{set}$ are computed by multiplying the reflectance spectra of the color chips with the illuminant spectrum (*L* for instance here).

Spectral reconstruction of *L* can be written [18]:

(14) $$\hat{L}=T_{set}T_{set}^{T}F{\left(F^{T}T_{set}T_{set}^{T}F\right)}^{-1}F^{T}L$$

$T_{set}T_{set}^{T}F{\left(F^{T}T_{set}T_{set}^{T}F\right)}^{-1}F^{T}$ is an oblique projection operator. Unlike the intrinsic reconstruction, $\hat{L}$ is not a simple combination of the sensor’s spectra because of the influence of the prior radiance data on $R_{t}$. Instead, $\hat{L}$ belongs to an another space generated by a basis of extrapolated spectral sensitivities. Because of the reciprocity of the pseudo-inverse operation, these extrapolated sensitivities are contained in an $F_{t}$ matrix computed from the reconstruction operator $R_{t}$:

(15) $$F_{t}=R_{t}{\left(R_{t}^{T}R_{t}\right)}^{-1}$$

**Figure 8:**
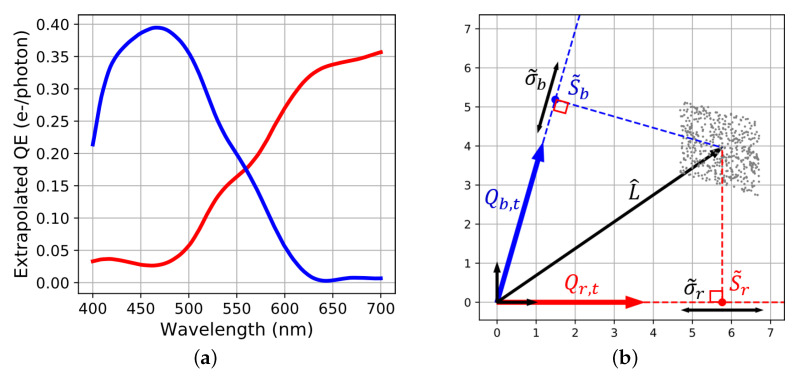
Linear regression over the X-Rite ColorChecker Classic (**a**) Extrapolated sensitivities $F_{t}$ obtained by calculating the pseudo-inverse of the reconstruction matrix. (**b**) Associated vector basis and spectral reconstruction in the sensor space. The orthogonal projections of signals and standard deviation $\tilde{S}$ and $\tilde{\sigma }$ are found with Equation (8) but using ${∥Q_{r,t}∥}_{2}$ and ${∥Q_{b,t}∥}_{2}$ instead of ${∥Q_{r}∥}_{2}$ and ${∥Q_{b}∥}_{2}$.

In turn, $F_{t}$ defined two axis called $Q_{r,t}$ and $Q_{b,t}$. The representation of $\hat{L}$ is given in Figure 8.

### 3.3. Tikhonov Regularization (Ridge Regression)

Both previous reconstructing operators come from inversion of the acquisition process. These inversions are known to amplify noise. A classical method to limit the increase of noise is to perform a Tikhonov regularization also known as ridge regression. Ridge reconstruction operator $R_{r}$ is written as:

(16) $$R_{r}=F{\left(F^{T}F+\alpha .I_{p}\right)}^{-1}$$

α is a real factor that controls the conditioning of the matrix, $I_{p}$ is the p×p identity operator. When α increases, the matrix becomes better conditioned so the inverse limits noise amplification. However spectral reconstruction operator gets biased [20]. As before, we can derive corresponding sensitivity functions or matrix $F_{r}$ as follows:

(17) $$F_{r}=R_{r}{\left(R_{r}^{T}R_{r}\right)}^{-1}$$

**Figure 9:**
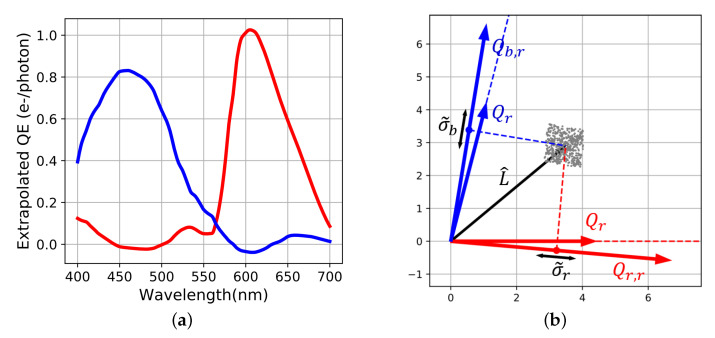
Tikhonov regularization (**a**) Extrapolated spectral sensitivities. (**b**) Spectral reconstruction with α=15. The orthogonal projections of signals and standard deviation ${\tilde{S}}_{i}$ and ${\tilde{\sigma }}_{i}$ are found with Equation (8) replacing ${∥Q_{r}∥}_{2}$ and ${∥Q_{b}∥}_{2}$ by ${∥Q_{r,r}∥}_{2}$.

Corresponding $Q_{r,r}$ and $Q_{b,r}$ are still linear combinations of $Q_{r}$ and $Q_{b}$, so they belong to the sensor’s space Figure 9.

Noise reduction comes from the modification of the shape of uncertainty volume. Noise surface sides are aligned with the axes and its size depends on α. Estimation of the optimal α coefficient is problem dependent and will not be discussed in this article [23,24].

### 3.4. Color Correction: Projection in a Color Space

**Figure 10:**
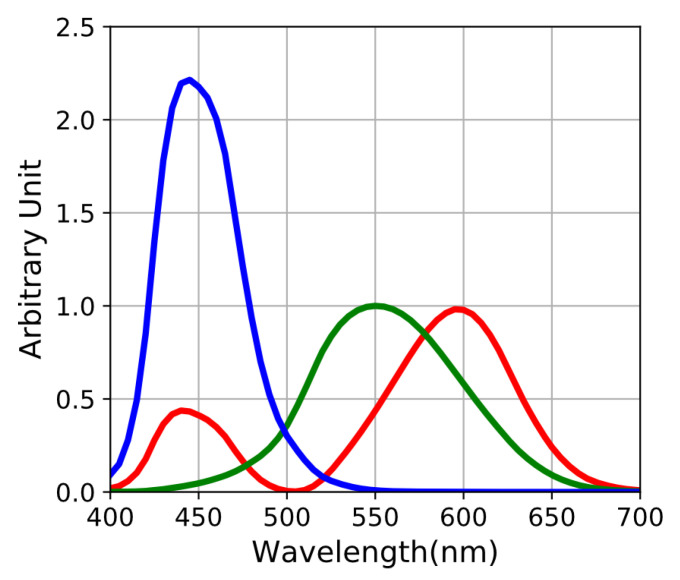
Color matching functions expressed to be used with radiances in photon flux units.

Color correction consists in transforming raw signal acquired by the sensor toward human tristimuli color data. There exist plenty of methods [11,25,26,27], among which linear color corrections matrix (CCM) is the most widely and extensively used. Spectral properties of the standard human observer are defined by CIE (*Commission internationnale de l’éclairage*) as the color matching functions (CMF) x(λ), y(λ), z(λ) (Figure 10). These curves taken as a n×3 matrix *H* allow to compute color data according to the CIE XYZ 1931 standard in the same way as sensor raw acquisition Equation (7). *H* is given by:

(18) $$H=\left(\begin{array}{ccc} x(\lambda_{1}) & y(\lambda_{1}) & z(\lambda_{1})\\ \vdots & \vdots & \vdots \\ x(\lambda_{n}) & y(\lambda_{n}) & z(\lambda_{n}) \end{array}\right)$$

*H* allows to compute a CCM [11]. As color coordinates are limited to CIE XYZ 1931 color space and CCM normalization is not performed, we actually call it $CCM_{kernel}$. This one is given by:

(19) $$CCM_{kernel}=H^{T}R=HF{\left(F^{T}F\right)}^{-1}$$

CCM contains an implicit spectral reconstruction operator *R* so geometrical interpretations given previously for radiance estimation remain valuable for color applications. *X*, *Y* and *Z* are color coordinates analogous to $S_{r}$ and $S_{b}$. They allow to compute the orthogonal projection of $\hat{L}$ on color matching axes (denoted $\tilde{X}$, $\tilde{Y}$ and $\tilde{Z}$) in the sensor’s vector space. To represent sensor and color axis at the same time on graphics, we show that applying $CCM_{kernel}$ is equal to apply the projection of *H* in the sensor’s space:

(20) $$\begin{array}{cc} \left(\begin{array}{c} X\\ Y\\ Z \end{array}\right) & =CCM_{kernel}F^{T}L\\ \; & =H^{T}F{\left(F^{T}F\right)}^{-1}F^{T}L\\ \; & ={\left(F{\left(F^{T}F\right)}^{-1}F^{T}H\right)}^{T}L\\ \; & ={\hat{H}}^{T}L \end{array}$$

**Figure 11:**
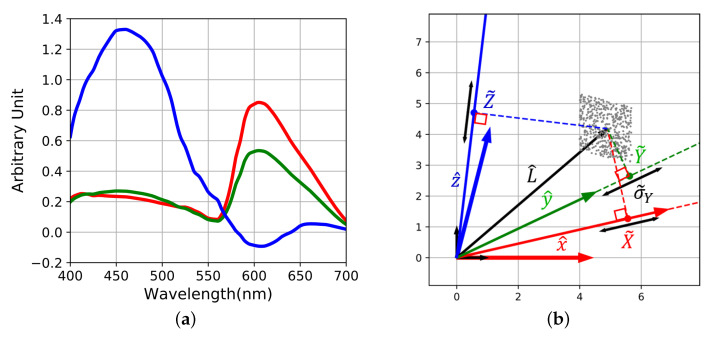
(**a**) Color matching functions projected into co-planar vectors $\hat{x}\hat{y}\hat{z}$ in sensor space, (**b**) corresponding geometry. Spectral noise is projected orthogonally on color matching axes giving $\tilde{X}\tilde{Y}\tilde{Z}$ and corresponding variances onto color matching channels.

As shown in Figure 11 the projection, $\hat{x}$, $\hat{y}$ and $\hat{z}$ only span two dimensions (because of the choice of a two channels sensor). The projection of the uncertainty volume of $\hat{L}$ produces uncertainties ${\tilde{\sigma }}_{x}$, ${\tilde{\sigma }}_{y}$ and ${\tilde{\sigma }}_{z}$. Amplitude of color noise depends on the size of the spectral uncertainty volume as well as its orientation regarding color matching axes.

This section provides some illustrations of how noise propagates from raw acquisition to spectral and color reconstructions. It shows that noise of output color images depends on many different aspects. From a physical point of view, first parameters are the raw sensitivities of the channels which impact raw noise levels. Then, inter-channel correlations have an impact on noise of spectral reconstruction by changing the size and shape of the uncertainty volume surrounding $\hat{L}$ (Figure 6). Finally, the noise of a color corrected image depends on one more criterion: the orientation of the target color space compared to the sensor axes (Figure 11). From a computational point of view, these noise levels depend also on the method used in spectral and color reconstruction.

## 4. Raw, Color and Contravariant Signal to Noise Ratios

Signal to noise ratio (SNR) is the measurement used to evaluate noise amplification in the literature. First, SNR on raw images can be computed independently over each spectral channel. Under the hypothesis of considering only photon shot noise and readout noise, the overall variance of a uniform image is $\sigma_{i}^{2}=\sigma_{ph}^{2}\left(\langle S_{i}\rangle \right)+\sigma_{rn}^{2}$. For the *p* channels of the sensor, raw SNR can be computed in decibels as:

(21) $$\begin{array}{cc} SNR_{i} & =20\log_{10}\left(\frac{\langle S_{i}\rangle }{\sigma_{i}^{2}}\right)=20\log_{10}\left(\frac{\langle S_{i}\rangle }{\sqrt{\sigma_{ph}^{2}\left(\langle S_{i}\rangle \right)+\sigma_{rn}^{2}}}\right)\\ i & =\{1,\ldots ,p\} \end{array}$$

As shot noise depends on the intensity, the SNR is greater when the signal increases. Inversely in low-light conditions SNR decreases [28]. Regarding geometrical representation of Figure 5, raw SNR can also be written:

(22) $$\begin{array}{cc} SNR_{i} & =20\log_{10}\left(\frac{{\tilde{S}}_{i}}{{\tilde{\sigma }}_{i}}\right)\\ i & =\{1,\ldots ,p\} \end{array}$$

This definition is valuable at the input of the processing chain. But noise propagation is classically evaluated by studying the SNR at the output image. These ones can be color images (three RGB channels) or multispectral (we consider it contains *n* channels corresponding to each wavelength).

**Figure 12:**
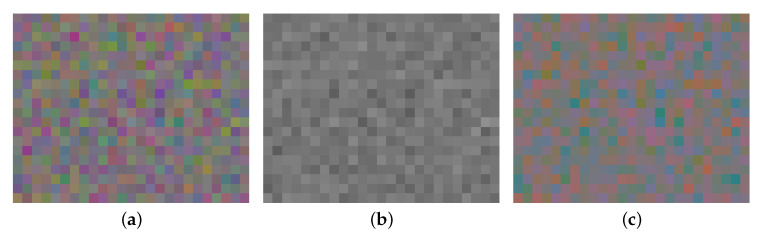
If a grey uniform scene is taken with a color camera under dim light (**a**), one can separate its luminance (**b**) to its chrominance (**c**).

In case of color images, each pixel contains a RGB triplet expressed in a display space (sRGB for example) [29]. As color is a perceptive entity, color SNR criteria must be representative of human perception [30,31]. Most standard color spaces are related to the standard of the *Commission Internationale de l’Éclairage* (CIE) spaces, namely CIE XYZ 1931. Color images can be separated into luminance and chrominance components (Figure 12). With sRGB display space, luminance is computed using RGB triplet values:

(23) Y=0.213R+0.715G+0.072B

Then, variances of RGB triplet are extracted from uniform area in output images (such as Figure 12). In practice, this evaluation is often performed over an achromatic (grey) uniform image. By propagating variances of RGB triplets (supposed to be statistically independent) through linear combinations, SNR over the luminance channel is computed as:

(24) $$\begin{array}{cc} \sigma_{Y}^{2} & =0.213^{2}\sigma_{R}^{2}+0.715^{2}\sigma_{G}^{2}+0.072^{2}\sigma_{B}^{2}\\ SNR_{Y} & =20\log_{10}\left(\frac{\langle Y\rangle }{\sigma_{Y}}\right) \end{array}$$

If we consider Figure 11, this noise can also be computed by:

(25) $$SNR_{Y}=\frac{\tilde{Y}}{{\tilde{\sigma }}_{Y}}$$

$SNR_{Y}$ associated to luminance is a convenient criterion because it is just a scalar. To be more accurate, one may also evaluate the impact of noise on chromaticity in other color spaces. For this evaluation, pixels are projected in the so called CIELAB color space [32] generating a cloud of points. Chromaticity noise can be represented as a volume in that space [2]. Because of non-linearity of the transformation between CIE XYZ 1931 and CIELAB we do not use this method in the following. Instead, we investigate chromaticity channels by applying Equation (25) on *X* and *Z* channels (see Figure 11).

Finally, for the case of multispectral images, SNR quantification is more challenging. The output image is a cube of data where the third dimension contains reconstructed spectra from each pixel over *n* wavelength samples. If spectra are linearly reconstructed, it is possible to evaluate SNR, at each wavelength sample, by using the covariance matrix of the reconstructing operator [2]. With that method, output SNR is expressed over *n* components, so it allows to draw standard deviation curves around the noise free spectral reconstruction which may be very convenient (as in Figure 19 in next section). Another way to illustrate SNR attached to multispectral data is to project reconstructed spectra in a color space and apply the same evaluation as for color images. SNR is, in that case, computed over the three components of the color space. These two ways of quantifying SNR in multispectral data have a common issue: their dimension. In fact, linear spectral reconstruction is an ill-posed problem where *n*-dimensional data are evaluated from *p*-dimensional ones (*p* is the number of channels of the sensor, usually lower than *n*). So linear reconstruction follows *p* degrees of freedom, then its SNR would be better represented over *p* dimensions instead of *n* or 3. To quantify SNR of spectral reconstruction over its real dimension, we introduce the contravariant SNR.

### 4.1. Definition of the Contravariant Signal to Noise Ratio

In spectral reconstruction, the estimated spectra $\hat{L}$ is a weighted sum of $Q_{r}$ and $Q_{b}$ (or their analogs if using training on dataset or Tikhonov regularisation). For a non-orthogonal basis, these weights are the contravariant coordinates of the vector $\hat{L}$ projected into the sensor basis [33]. In opposition, raw acquisition vectors correspond to the covariant coordinates of $\hat{L}$ in that basis. Contravariant coordinates are obtained by applying the metric tensor on covariant coordinates, they are often denoted with upper indexes following the so-called Einstein’s notation [33]:

(26) $$\begin{array}{cc} \left(\begin{array}{c} S^{1}\\ \vdots \\ S^{p} \end{array}\right) & ={\left(F^{T}.F\right)}^{-1}.\left(\begin{array}{c} S_{1}\\ \vdots \\ S_{p} \end{array}\right)\\ \; & =\left(R^{T}.R\right).\left(\begin{array}{c} S_{1}\\ \vdots \\ S_{p} \end{array}\right) \end{array}$$

**Figure 13:**
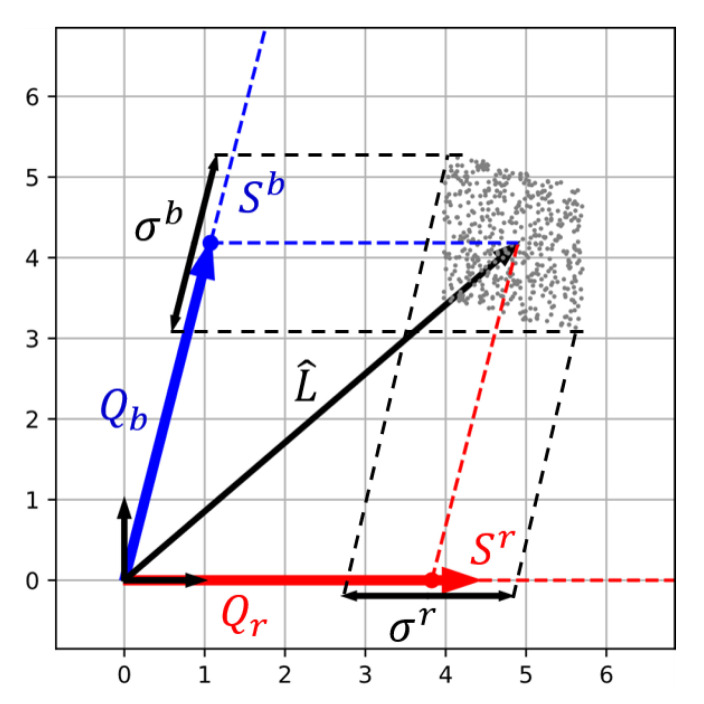
Projection of the uncertainty volume onto contravariant coordinates. Uncertainty in spectral reconstruction is given by contravariant noise $\sigma^{r}$ and $\sigma^{b}$. When the angle between channel is smaller, the contravariant coordinates and SNR decrease. Spectral reconstruction becomes “more noisy”.

A geometrical illustration is given in Figure 13 for the previous red and blue sensor. In the same way as $\hat{L}$, uncertainty volume associated to spectral reconstruction can be projected alongside axes. As a result, we get the uncertainties of contravariant coordinates of $\hat{L}$ denoted $\sigma^{r}$ and $\sigma^{b}$. These uncertainties constitute what we call in the following: contravariant noise. Contravariant signals and noise definitions allow to compute a contravariant SNR associated to each dimension:

(27) $$\begin{array}{cc} SNR^{i} & =20.\log_{10}\left(\frac{S^{i}}{\sigma^{i}}\right)\\ i & =\{1,\ldots ,p\} \end{array}$$

Contravariant noise remains valuable when the training method is used as well as the ridge regression. The metric tensor that must be applied in Equation (26) is then, respectively, $\left(R_{t}^{T}.R_{t}\right)={\left(F_{t}^{T}.F_{t}\right)}^{-1}$ or $\left(R_{r}^{T}.R_{r}\right)={\left(F_{r}^{T}.F_{r}\right)}^{-1}$. Therefore, additionally to evaluate the impact of the sensor’s metric, contravariant noise can be used to compare the influence of several reconstructing methods.

### 4.2. Example of Contravariant SNR Evaluation to Compare Several Spectral Reconstructing Methods

Using contravariant SNR, we can compare noise propagation through several reconstruction methods (intrinsic, regression, ridge regression with two different α parameters). The sensor is still the two-dimensional one and the spectrum to be reconstructed is still *L* (uniform photon distribution over wavelength). The spectral reconstruction accuracy is measured in terms of angular error. The exposure parameter for raw computation remains the same as Section 3.

**Figure 14:**
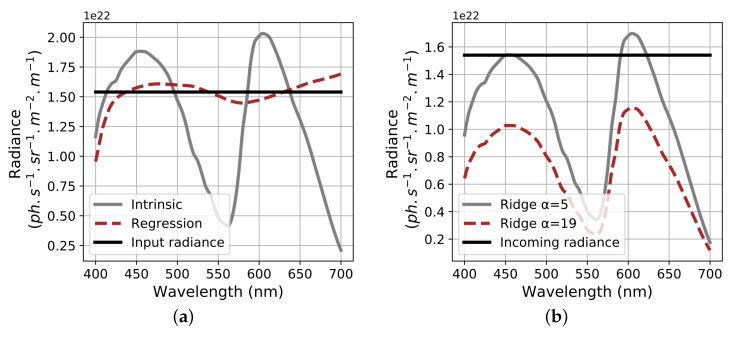
(**a**) Input radiance with corresponding reconstruction for intrinsic and regression methods, (**b**) reconstruction using ridge regression with two different α parameters.

The angular error between *L* and $\hat{L}$ is given in Table 2, it is computed similarly to Equation (5). Corresponding graphical noise free reconstructions are displayed Figure 14.

Table 2 shows that the most accurate spectral reconstruction of *L* is obtained using the linear regression method which confirms visual results of (Figure 14). It also shows that the bias induced by the ridge regression operator is negligible in terms of angle. This occurs because the angle between the red and blue channels is wide enough. So using intrinsic or ridge regression does not change significantly the spectral distribution of the reconstructed spectrum.

**Figure 15:**
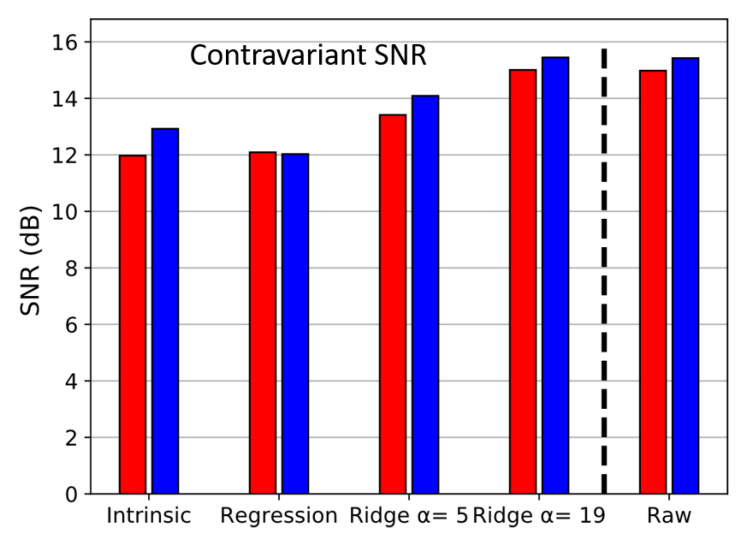
Contravariant signal to noise ratio (SNR) corresponding to several reconstructing methods for over red and blue channel (colors of the bars).

Joint evaluation of contravariant SNR for each method has been computed in Figure 15. Their values are close to each other even if regression method appears to be the most noisy one. On the contrary, higher SNR are obtained with ridge regression for increasing α, confirming the interest of ridge regression in noisy conditions.

## 5. SNR Analysis of Various Common Sensors

We perform a benchmark of several sensors having different spectral configurations. The goal is to identify a configuration which provides the lower noise amplification across color correction or spectral reconstruction. In accordance with previous investigations, we analyze SNR at raw, spectral reconstruction and color correction steps. We compare six different sensors called respectively RGB, CMY, RGBW, RGBWir, RYB and RGBC.

- RGB and CMY are constituted of respectively red, green, blue and cyan, magenta, yellow channels, they are classical designs for color image sensors.
- RGBW and RGBWir contain a white pixel (no color filter) more sensitive than others. RGBWir do not own infrared cutoff filter, so compared to RGBW sensor, it acquires light from both visible near-infrared domains. Both sensors have been proposed for low light conditions.
- RYB has a yellow channel twice sensitive as red and blue.
- RGBC owns a cyan channel additionnally to RGB also twice sensitive.

**Figure 16:**
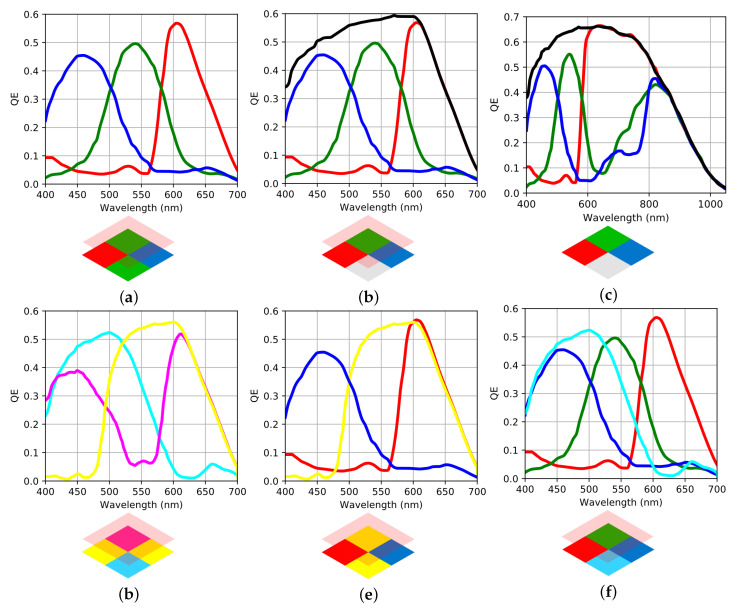
Spectral sensitivities of each sensor. Color code correspond to the nature of spectral sensitivity of the channels. (**a**) RGB, (**b**) RGBW, (**c**) RGBWir, (**d**) CMY, (**e**) RYB, (**f**) RGBC.

Spectral sensitivity curves are mostly proprietary data. So the benchmark is carried out on theoretical built sensors based on one unique model taken from the Teledyne-E2V Onyx EV76C664 sensor. Electronical properties are given in Table 3. Sensitivity curves of RGBWir are those from Onyx [34]. RGB, and RGBW are built applying an infrared cutoff filter in front of RGBWir channels. Similarly, CMY channels are built using the white sensitivy of Onyx, with Fuji CMY color filters in front [35]. Final spectral sensitivites are given in Figure 16.

The benchmark starts with an evaluation of noise free performances of the sensor (spectral and color reconstruction). For each sensor, spectral channels are equally considered for the computation of the spectral reconstruction operator. This one is computed using (Equation 11) which do not imply any training set (to avoid bias due to prior data). So *R* is a n×p operator where p={3,4} depending on the number of channels. Spectral accuracy is evaluated over the equal distribution radiance spectrum *L*. The accuracy is given in angle as in Table 2, so the results of the benchmark are given in Table 4.

Color performances are checked over the X-Rite ColoChecker Classic using the $\Delta E_{76}$ criterion (Table 5). For coherence CCM is computed with Equation (19), as well as to avoid bias due to prior data (still no training set used to compute the CCM).

Table 5 shows that studied sensors have comparable color performances. Nonetheless, RGBWir sensor gets the poorest color performance due to the near-infrared part of the signal [36].

Second step of the benchmark is to investigate noise propagation. This study is performed for both high and low light level. For each sensor, we simulate a noisy acquisition of the *L* spectrum under controlled exposure setup (Table 6). First, illumination is set to 10 lux which is a dim light for human vision. However as the sensor is very sensitive due to large pixels, these conditions are those of high light level.

Illumination level and aperture are fixed, integration time is computed such that acquisition reaches the maximum value without saturating any channel of any sensor. For information, the saturation integration times of each sensor are given Table 7.

**Figure 17:**
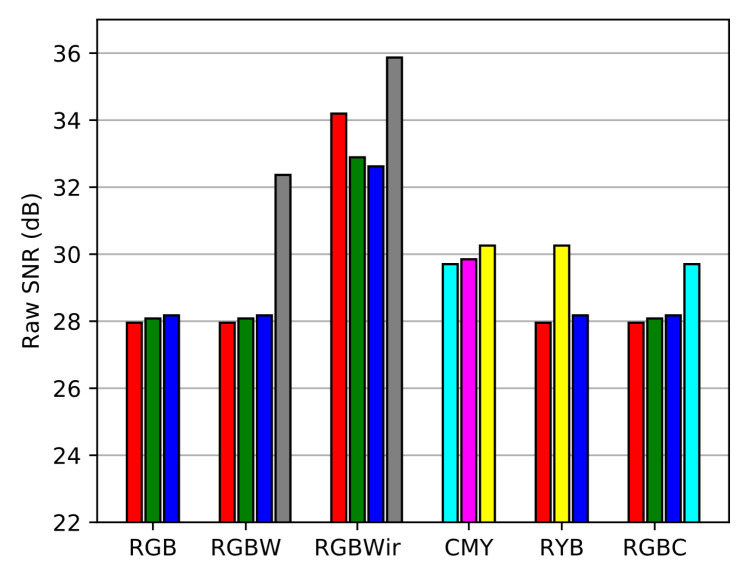
SNR over raw channels of each sensors, the colors code follows the name of the channels.

Raw SNR is computed according to the definition of Equation (21). This SNR evaluation is performed for each channel of each sensor and is represented Figure 17.

**Figure 18:**
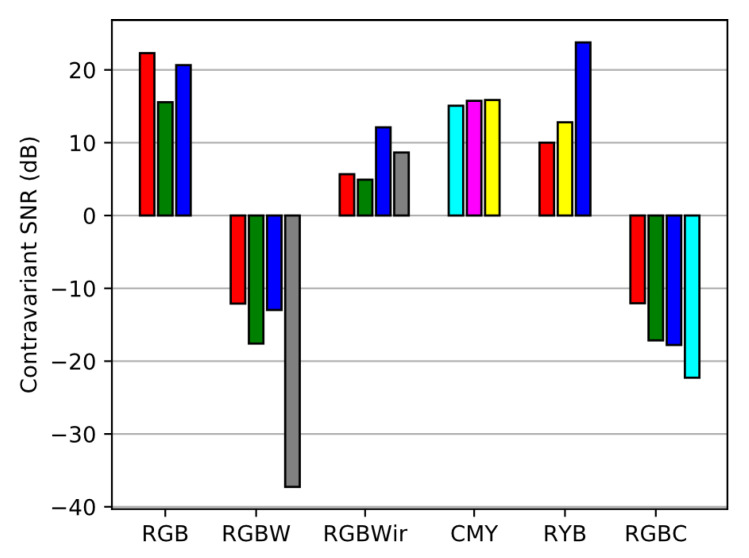
Contravariant SNR over channels of the sensors.

As expected, sensors with wider spectral sensitivities get higher raw SNR than others since they contain more sensitive channels. So the classical RGB is the sensor that owns the lowest raw SNR. Then we compute the contravariant SNR (Equation (27)) to investigate the spectral reconstruction level as shown in Figure 18).

**Figure 19:**
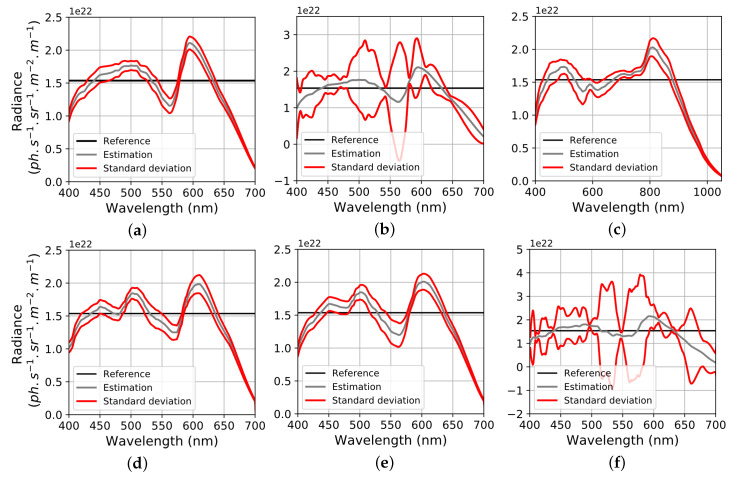
For each sensor, intrinsic spectral reconstruction with upper and lower standard deviations wavelength by wavelength (red curves are similar to standard deviation error bars). (**a**) RGB, (**b**) RGBW, (**c**) RGBWir, (**d**) CMY, (**e**) RYB, (**f**) RGBC.

The result displays negative contravariant SNR. This occurs when the absolute value of the contravariant signals are lower than their noise level. In case of negative SNR, results of spectral reconstruction cannot be exploited because they are too noisy. Moreover, contravariant SNR almost follow the inverse trend than raw SNR. Indeed, in sensors with wide spectral channels, inter-channel correlations are increased and cause a high noise amplification at spectral reconstruction level (Figure 6). In Figure 19, we illustrate the results of noisy spectral intrinsic reconstructions (at same exposure setup) wavelength by wavelength. This figures clearly shows the difficulty of RGBW and RGBC to reconstruct spectra from noisy signals. This happens because respectively W and C channels are too correlated to RGB channels.

**Figure 20:**
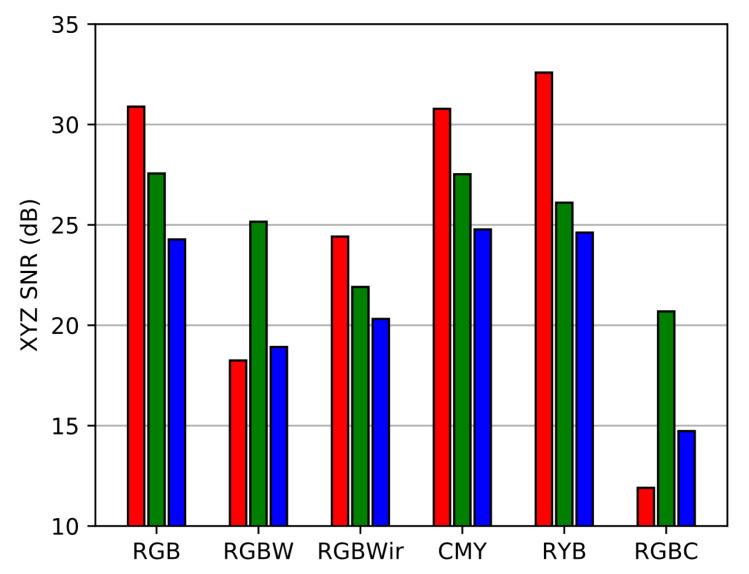
SNR over color components of each sensor.

**Figure 21:**
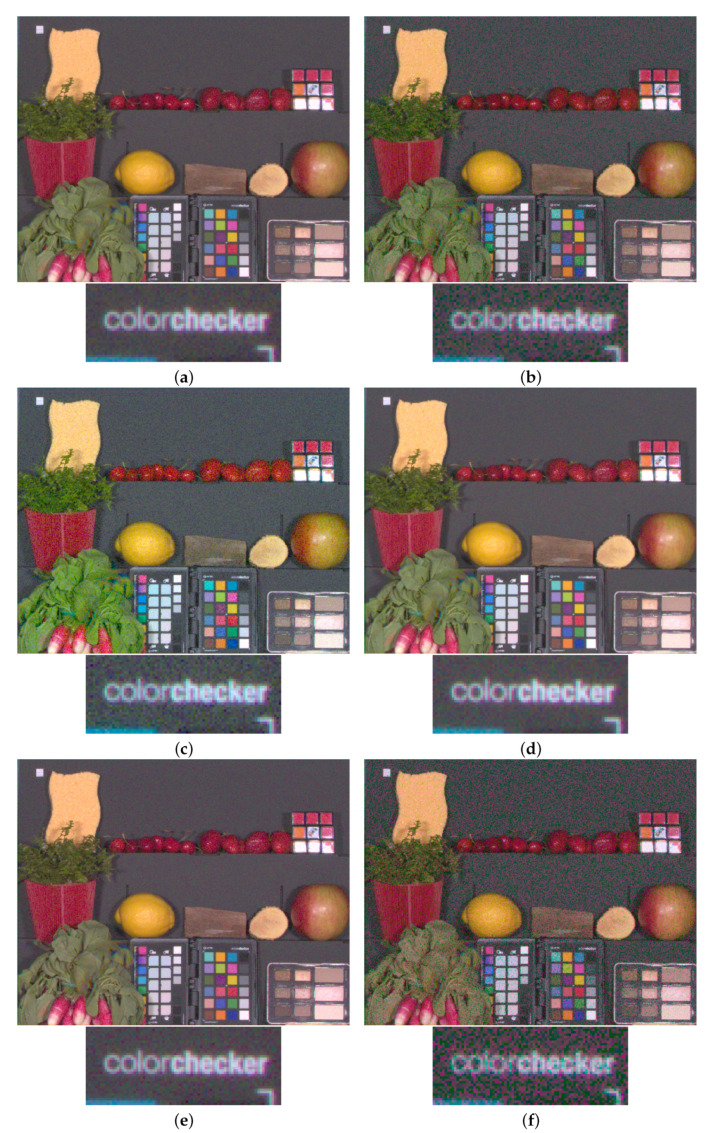
Illustration of color image renderings for each sensor after applying the CCM on raw data. Each frame is displayed with a zoom around the “colorchecker” word on the X-Rite ColorChecker Passport Photo. (**a**) RGB, (**b**) RGBW, (**c**) RGBWir, (**d**) CMY, (**e**) RYB, (**f**) RGBC.

The final step of the benchmark is an SNR evaluation after color correction. On Figure 20, we display SNR over *X*, *Y* and *Z* components, enlarging the usual picture of $SNR_{Y}$ comparison (see Equation (25)). Here we see that color SNR follows the trend of contravariant SNR. To complete this high light level benchmark, we use the simulating framework of Section 2 to simulate the noisy color renderings over realistic images corresponding to the physical setting of the benchmark Figure 21. Once again, images are computed with high resolution multispectral images from the ReDFISh dataset [13]. For each sensor, color images have been computed with a normalized update of the $CCM_{kernel}$ whose calculus is based on Equation (19) such that no training set is used.

**Figure 22:**
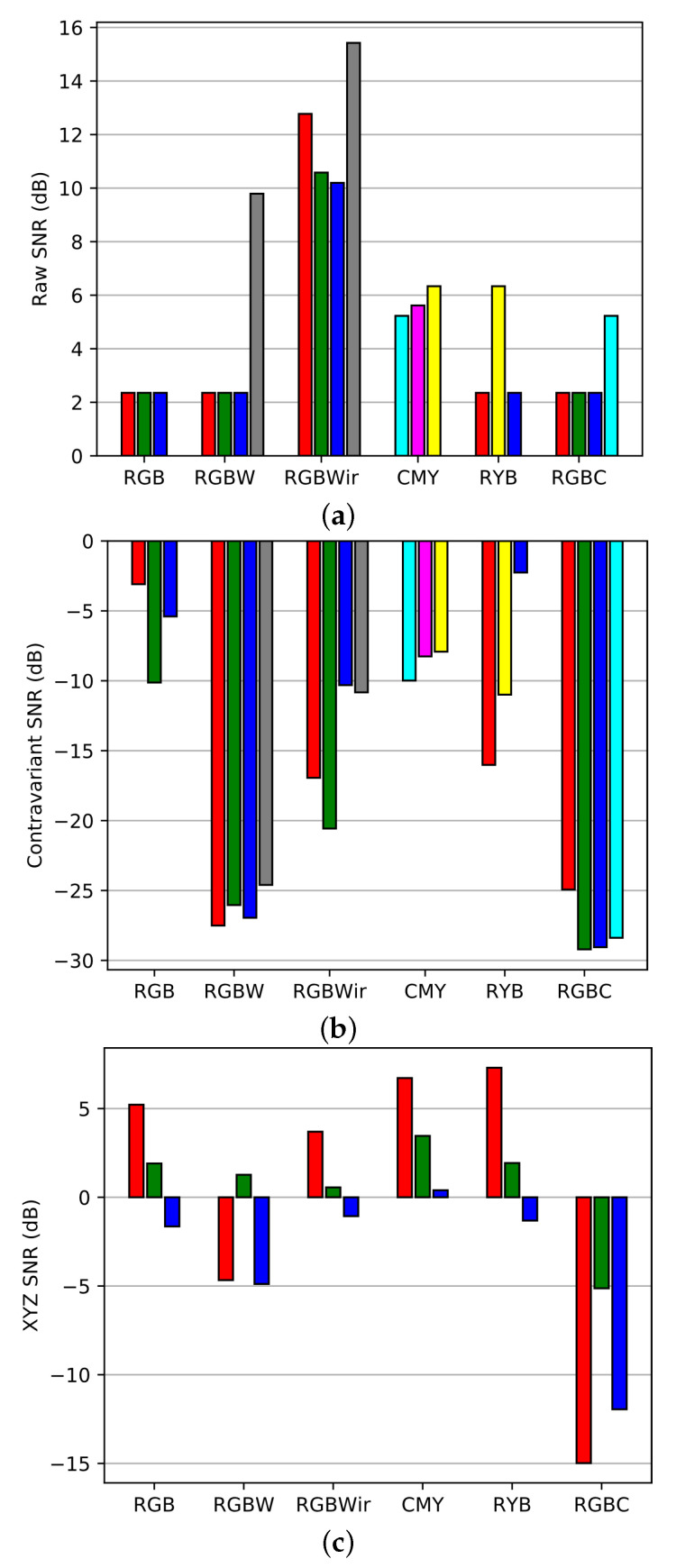
SNR results for dim light conditions. (**a**) SNR raw, (**b**) contravariant SNR, (**c**) color SNR.

In a second test, we perform the same benchmark but in dim light conditions. All physical parameters remain equal except the illumination factor which is divided by a 50 factor: $N_{lux}$ = 0.2 lux. Histogram results are displayed in Figure 22 for each type of SNR.

We can draw some conclusions from this evaluating section. For both high and low signal levels, higher raw SNR are obtained for most sensitive channels. Contravariant SNR evaluation shows that reconstructing spectra from sparse raw data is very sensitive to noise and could not be performed in low light conditions (negative contravariant SNR). Moreover, contravariant SNR seems to remain higher in both conditions for the RGB sensor. This shows that spectral reconstruction is mostly sensitive to inter-channel correlation instead of raw sensitivity. Finally, color SNR of the RGB sensor are overtaken by CMY and RYB at low light level. This change in the tendencies of the benchmark are due to the readout noise impact which becomes higher in low light level. So for color applications, a smart compromise must be found between raw sensitivity, inter-channel correlation and sensor/color axes orientations to minimize noise in output images. RGBW still gets bad results in low light conditions because of high correlations of W and the RGB spectral space. So from a spectral point of view, the W channel does not have benefit. However, we did not perform pansharpening [6] in this study, so the interest of W channel is not highlighted here.

## 6. Conclusions

This paper was focused on the impact of the spectral sensitivities of imaging sensors on noise of output image (color and multispectral). To perform our analysis, we studied the color correction step of image processing and discarded all other steps like denoising or demosaicking. So we started introducing an appropriate simulating framework to display an example of noise amplification through color correction.

Then, we proposed a novel geometrical way of representing noise propagation from raw acquisition through spectral reconstruction and color correction. This approach considers spectra as vectors [10] whereas noise is represented as uncertainty domains (segments or volume) around the noise free position [2]. We showed that noise quantity in the final color image depends on three main spectral dependencies: raw sensitivity, inter-channel correlations, relative orientation of sensor and color spaces. Based on our approach, we also were able to represent spectral reconstruction of other methods like least square minimization over a training set and Tikhonov regularization.

After recalling computation of raw and color signal to noise ratios to quantify noise in uniform images, we introduced the contravariant SNR to quantify noise in spectral reconstructions. Compared to other quantifiers, this one is accurate in terms of dimension. We also generalized it such as it can be computed when using the different linear reconstructing methods.

Finally, using the different SNR defined in the article, we evaluated spectral and color performances of several sensors with spectral characteristics close to commercial products. We found out that the minimization of noise in output color images needs a fine compromise between three spectral dependencies we highlighted in Section 3. However, having low inter-channel correlations seems to be mandatory if the goal is to reconstruct spectra, which is shown by the results of the RGB sensor in terms of contravariant SNR.

The next step of this work is to use the description drawn in this paper to optimize spectral sensitivities and make image acquisition less noisy. Additionally to what already exists [16,18], we would like to find a geometrical method based on the presented geometrical framework to find an optimum sensor. We also can expect that this formalism would help one to optimize methods of spectral reconstruction or color correction.

## Figures and Tables

**Table 1:** Physical setting of the simulation.

Sensor and Exposure Parameters	Values
Pixel pitch $a_{pix}^{2}$	10 (μm)
Readout noise $\sigma_{rn}$	10 (e-)
Illumination level $N_{lux}$ (in lux)	10 lux
$f_{\#}$	1.8
integration time ($t_{i}$)	4.4 ms
CVF	$\frac{1}{10000}$

**Table 2:** Angular error between reference radiance spectrum and reconstructed spectra (the lowest, the best).

Reconstructing Method	Intrinsic	Regression	Ridge α=5	Ridge α=19
$\theta_{L,\hat{L}}$ (in ${}^{\circ }$)	21.82	4.14	21.83	21.85

**Table 3:** Physical parameters of the sensor.

Sensor’s Parameters	Values
$Q_{sat}$	10,000 (e-)
Pixel pitch	10 (μm)
Readout noise	10 (e-)

**Table 4:** Angular error between constant radiance and its intrinsic spectral reconstruction for each sensor.

	RGB	RGBW	RGBWir	CMY	RYB	RGBC
$\theta_{L,\hat{L}}$ in (${}^{\circ }$)	16.27	15.93	20.15	15.29	15.76	15.89

**Table 5:** Color performance of the sensor over the 24 patches of the X-Rite ColorChecker Classic. No training set have been used for the computation of color correction matrix (CCM).

Color Error	RGB	RGBW	RGBWir	CMY	RYB	RGBC
$min(\Delta E_{76})$	0.34	0.14	0.18	0.32	0.14	0.32
$mean(\Delta E_{76})$	3.23	2.81	10.94	2.81	3.33	3.98
$max(\Delta E_{76})$	11.19	9.00	60.94	8.42	8.88	10.58

**Table 6:** Exposure parameters.

Parameters	Values
Illumination ($N_{lux}$)	10 lux
$f_{\#}$	1.8
integration time ($t_{i}$)	11 ms

**Table 7:** Integration time when the most sensitive channel starts to saturate.

Integration Time at Saturation	RGB	RGBW	RGBWir	CMY	RYB	RGBC
$t_{i}^{sat}$ (in ms)	58	24	11	37	37	42
